# Transport to School and Mental Well-Being of Schoolchildren in Ireland

**DOI:** 10.3389/ijph.2021.583613

**Published:** 2021-04-09

**Authors:** András Költő, Aoife Gavin, Colette Kelly, Saoirse Nic Gabhainn

**Affiliations:** Health Promotion Research Centre, School of Health Sciences, National University of Ireland Galway, Galway, Ireland

**Keywords:** cycling, children, health behaviour in school-aged children, HBSC, mental well-being, school, active transport

## Abstract

**Objectives**: We explored whether modes of transport (cycling, walking, public transport or private vehicle) between home and school are associated with mental well-being in children aged 10–17 years, participating in the Irish Health Behaviour in School-aged Children (HBSC) study.

**Methods**: Scores on the World Health Organization Well-being Index and the Mental Health Inventory five-item versions, self-reported life satisfaction, happiness with self, body satisfaction, excellent self-rated health, and multiple health complaints of 9,077 schoolchildren (mean age: 13.99 ± 1.91 years, percentage girls: 52.2%) were compared across modes of transport, unadjusted and adjusted for gender, age, family affluence and area of residence.

**Results:** Those who reported using public transport reported poorer mental well-being than those using other means of transport, but adjusting for sociodemographic variables obscured these differences. The only exception was excellent health, where children who cycled outperformed the other three groups, even after adjustment for sociodemographic variables.

**Conclusions:** Cycling can improve well-being in children. However, in promotion of cycling, social and environmental determinants and inequalities which influence adolescents’ and their parents’ decisions on modes of transport, need to be considered.

## Introduction

The importance of regular physical activity for promoting both physical and mental well-being among children and adolescents is widely recognized (1, 2). Active school transport can provide a significant source of physical activity, and can enable children to meet the WHO guidelines of 60 min of physical activity per day (3, 4). Modes of active transport between home and school – walking and cycling – have been associated with health benefits and give a sense of increased independence to young people (5). Thus, it has been argued that they should be prioritized over transport by motor vehicle. Active transport is one of the priority areas within the Physical Activity Strategy for the WHO European Region 2016–2025 (6). The National Physical Activity Plan for Ireland (7) recognizes the many benefits of active transport beyond immediate physical activity gains and aims to develop walking, cycling and general recreational and physical activity infrastructure. In Ireland, the Green Schools initiative encourages schools to have an active travel plan that encourages pupils to use alternatives to a car, and supports and promotes active school transport (8).

Studies on the health-related impacts of children’s active travel have primarily focused on physical well-being and health determinants. There is clear and consistent evidence that children and adolescents who engage in active school transport report improved physical health such as lower body weight, healthier body composition, and better cardiovascular outcomes, including lower blood pressure and cholesterol levels (1, 3, 9).

There is a large body of empirical evidence highlighting the benefit of physical activity to young people’s mental well-being (e.g., 10, 11). However, there is a lack of evidence measuring the impact of active travel on the mental well-being of young people (12), particularly in Ireland. A recent scoping review of children and adolescent’s active travel in Ireland identified 19 studies exploring active travel, however none of these included mental well-being outcome measures (3). A limited number of international studies have found positive associations between active school transport and measures of mental health or mental well-being. One study in China reported that children in grades 4–12 (no age range reported) who engaged in active travel were less likely to report depressive symptoms as assessed by the Children’s Depression Inventory, when compared to children who did not (1). A study examining psychological well-being among Austrian children from 3rd and 4th grade (mean age: 9.6) found that active travel modes (walking, cycling and scooter) were associated with higher psychological well-being than passive transportation (using a car as a passenger or using public transport), assessed by visual analogue scales on their mood during the first and the last school lessons (12). Children cycling to school reported the highest psychological well-being, and children in general had a very positive attitude to cycling.

Active school transport is influenced by multiple health determinants, specifically: individual (age, gender), social (family, friends) and environmental factors (infrastructure, roads) (13). Sun et al. (1) reported that children from rural areas were more likely than urban children to choose active transport to school. Children from families with low socio-economic status were most likely to report walking, while children with high socio-economic backgrounds were most likely to report passive transportation.

The National Cycle Policy Framework in Ireland (14) acknowledges the importance of these determinants. It contains many objectives which aim to improve cycling through making changes in contextual factors, such as providing and maintaining cycling-friendly roads, ensuring that cycling and public transport systems are integrated, and improving driver education and standards in a way that private vehicle drivers observe the safety needs of cyclists. A study of barriers and promoters of active travel among school-aged children in Ireland (15) found that children from urban and disadvantaged schools were more likely to have actively traveled to school. Proximity to the school was the most frequently reported determinant that influenced active travel. In countries with a low prevalence of cycling, adolescent girls are much less likely to use a bicycle than boys, thus indicating that gender may have an impact on active school transport behaviours (5).

These findings from the literature suggest that a wide range of factors influence active travel in a complex way. The association of active school transport and mental well-being may be influenced by such determinants, including family affluence or area of residence (1, 16, 17).

This study examines the modes of transport to or from school and their associations with various dimensions of mental well-being of school-aged children in Ireland. While we were unable to find sufficient previous research to set specific hypotheses, we anticipated that commuting to and from school by cycling and walking would be associated with better mental well-being outcomes and that these associations would be influenced by sociodemographic factors such as gender, age, family affluence and area of residence.

## Methods

### Sample

We used data from a subsample of 9,077 children participating in the nationally representative 2018 Irish Health Behaviour in School-aged Children (HBSC), a World Health Organization collaborative cross-cultural study (age range: 10–17 years, mean age: 13.99, SD = 1.91). HBSC is an observational, cross-sectional epidemiological study of adolescent health and its psychosocial determinants. The study instrument was a paper-based questionnaire that participating children completed during school hours. The survey was carried out in adherence to the international HBSC study protocol (18) and was approved by the Research Ethics Committee of the National University of Ireland Galway. Informed consent was obtained from all participating students as well as their parents/guardians and school Principals. No reimbursement was offered or provided for participation. Children were informed that they are free not to answer any questions in the survey and to withdraw their participation at any time.

**FIGURE 1:**
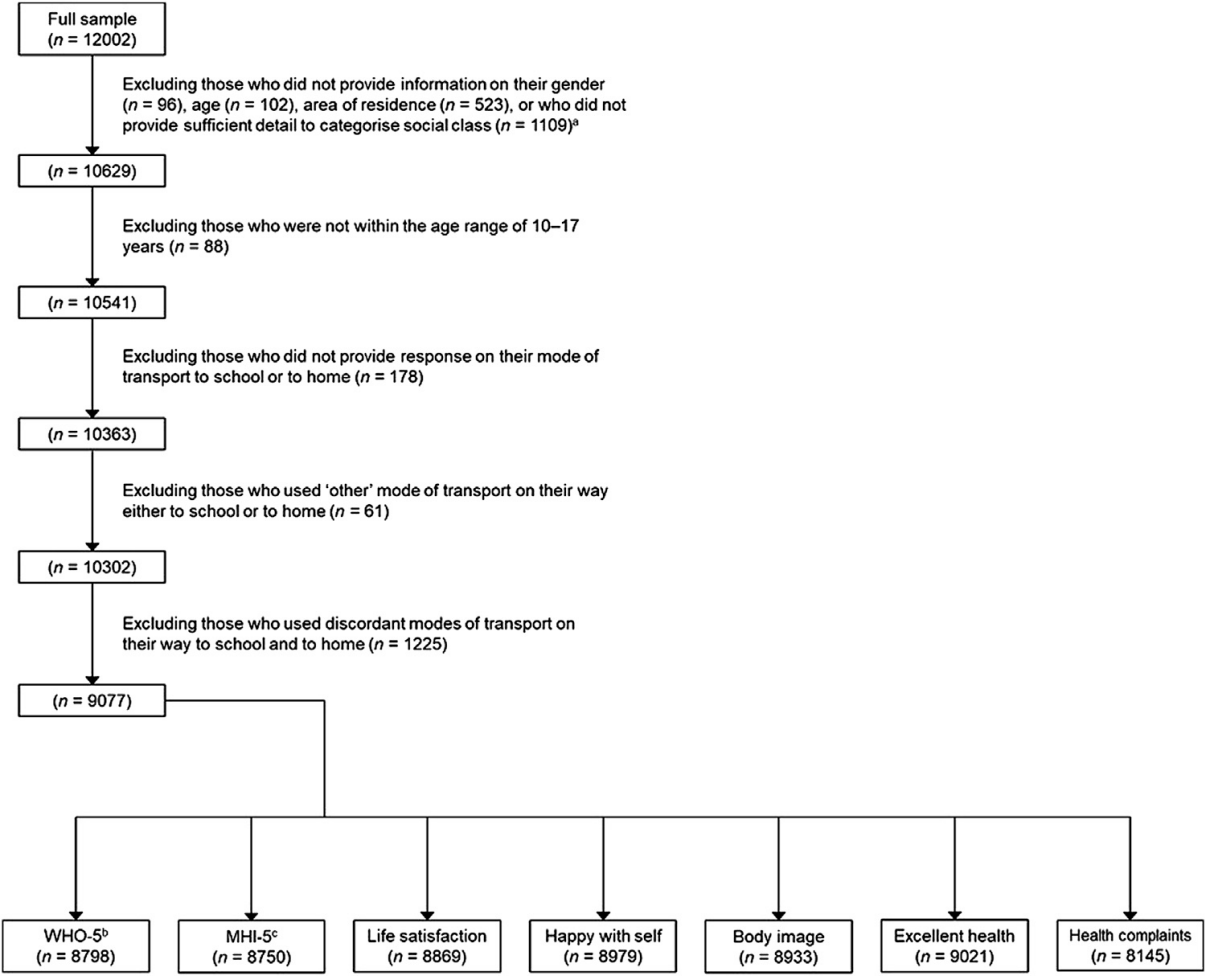
Sample selection flowchart, Health Behaviour in School-aged Children study in Ireland, 2018. ^a^There are some overlaps in the missing responses. ^b^World Health Organization Five-item Well-Being Index. ^c^Five-item Mental Health Inventory.

The entire sample of students in Class 5–6 of primary schools and Years 1–5 of post-primary schools contained data of 12,002 children. We have used a five-step procedure outlined in Figure 1 to select children for the present analyses. Children included in the final sample were those who provided information on their gender, age and area of residence; provided sufficient detail to categorize them into family affluence groups; were in the age range of 10–17 years; responded to questions on mode of transport to and from school (excluding ‘other’ way – see *Mode of Transport* Section); and whose travel to and from school were by the same mode. Many children gave different answers about their travel to and from school (*n* = 1,225); some reported that they went to school by private vehicle but walked home (*n* = 563, 46.0%), or walked to school but returned home by private vehicle (*n* = 136, 11.1%). Other substantial subgroups reported they were driven to school and went home by public transport (*n* = 268, 21.9%) or used public transport on their way to school and returned home by a private vehicle (*n* = 118, 9.6%). These patterns raised the potential for confounding during the testing of the relationships between modes of transport and mental well-being outcomes. Therefore we excluded children who reported discordant modes of transport to and from school. This selection procedure resulted in a subsample of 9,077 children. Missing data on mental well-being measures led to differences in the sample sizes in each analytic model described below (see Figure 1).

### Measures

#### Sociodemographic Variables

*Gender* of children were assessed with one item: “Are you a boy or a girl?“, with response options ‘a boy’ or ‘a girl’. Their *age* was derived from the year and month they were born, and the time of data collection. Comparative socioeconomic status was indicated by the Family Affluence Scale (FAS), a six-item composite measure developed by the international HBSC Network. The FAS contains items on different aspects of family wealth, including material belongings (how many cars and computers does the family own; whether there is a dishwasher in the home), housing (whether the respondent have their own bedroom; number of bathrooms in the family home), and non-essential expenditures (number of family holidays abroad last summer) (19). Absolute FAS scores (0–13) were transformed into a ridit-based *relative family affluence* score, which classified families belonging to the lowest 20%, medium 60% and highest 20% of relative family affluence (20). *Area of residence* was assessed by the item “Where do you live?“, with response options ‘city’, ‘town’, ‘village’, and ‘country’. In Ireland, status of cities and towns are legally designated. ‘Village’ refers to a compact settlement of houses. ‘Country’ (i.e., ‘countryside’) refers to areas where rural population resides, normally in individual homes separate from one another. The Central Statistics Office of Ireland defines these as having a settlement size of less than 1500 people (21).

#### Mode of Transport

Children were asked what mode of transport they use for the main part of their journey to and from school on a typical day. The response options were ‘walking’, ‘using a bicycle’, ‘using public transport (bus, train, tram or boat)’, ‘using a private vehicle (car, motorcycle or moped)’, or ‘other means’. As outlined above in the Sample section, we analyzed data of those children who provided concordant answers to both items (except ‘other means’). We have combined their responses for to and from school into one *mode of transport* variable.

#### Continuous Mental Well-Being Variables

The measure of *perceived well-being*, the World Health Organization Five-item Well-being Index (WHO-5) (22) contains items such as feeling ‘calm and relaxed’, or waking up feeling ‘fresh and rested’. Respondents marked their agreement with the items on a six-point Likert-type scale ranging from ‘At no time’ to ‘All of the time’ (within the last two weeks). The raw scores were transformed to a scale ranging from 0 to 100, where higher scores reflected better well-being. The scale had high internal consistency in our sample (Cronbach’s alpha = 0.88). *Mental health problems* were assessed by the Five-item Mental Health Inventory (MHI-5) (23). This scale contains both negatively and positively phrased items (e.g., during the last month the respondent felt ‘downhearted and blue’ or had been a ‘happy person’). Agreement with the items were indicated on a six-point Likert-type scale, ranging from ‘All the time’ to ‘None of the time’. Raw scores were transformed to a scale ranging from 0 to 100, where higher scores reflected poorer mental health. The scale showed high internal consistency in our sample (Cronbach’s alpha = 0.81). The Cantril ladder was employed as a measure of global *life satisfaction*, where ‘10’ indicates the best and ‘0’ the worst possible life (24). Mean scores on these variables were calculated for each of the transport groups.

#### Dichotomous Mental Well-Being Variables

As a measure of global self-esteem, children were asked whether they have been *happy with the way they are*. Children reporting ‘always’ or ‘very often’ were classified as being happy with themselves, while children reporting ‘quite often’, ‘seldom’, or ‘never’ were classified as not being happy with themselves. Children reporting that their body is ‘about the right size’ were classified as being *satisfied with their body* and were contrasted with those who stated that their body is ‘a bit too thin’, ‘much too thin’, ‘a bit too fat’ or ‘much too fat’. Self-rated health was classified into ‘excellent’ health and contrasted with ‘good’, ‘fair’, or ‘poor’ health. Children were asked to report the frequency of having eight somatic (e.g., headache, stomach-ache) and psychological (e.g., feeling low, feeling nervous) health complaints in the previous six months. Children who reported two or more health complaints more frequently than weekly within this time frame were classified as reporting *multiple health complaints* and were contrasted with those reporting fewer than two psychosomatic complaints with the same frequency.

The continuous and dichotomous outcome variables are further described in the Irish (25) and international (26) HBSC study reports.

### Statistical Analysis

Analyses were carried out in SPSS 24.0 (IBM Corp., Armonk, New York, United States). Associations of mode of transport with the sociodemographic variables and mental well-being outcomes were tested by analysis of variance (ANOVA) and covariance (ANCOVA) for the continuous variables and Chi-square tests for the binary variables (Table 1). The dichotomous outcome variables were further investigated by binary logistic regression. Following univariate tests, analyses were adjusted for gender, relative family affluence and area of residence as predictors and age as a covariate. The multivariate ANCOVA models were built in an iterative fashion to include significant predictors and interaction parameters. Transport groups were compared using post-hoc tests with Sidak adjustment. In the binary logistic regression models, cyclists were used as reference group. The contribution of the predictor variables were examined by Wald Chi-Square tests. Crude odds ratios (COR) were obtained to assess whether the other transport groups have different outcomes than those of the cyclists. The crude odds ratios were subsequently adjusted for gender, relative family affluence and area of residence (AOR). For all odds ratios, 95% confidence intervals (CI) were calculated. The effect of interactions was not tested. Model fit was verified. No multicollinearity was detected in the predictor variables. Statistical significance for all analyses was defined as *p* < 0.05.

**TABLE 1:** Descriptive statistics – sociodemographic variables and binary mental well-being outcome variables, Health Behaviour in School-aged Children study in Ireland, 2018.

	Total	Cycling	Walking	Private vehicle	Public transport	*p*
		*n* (%)	*n* (%)	*n* (%)	*n* (%)	
Gender	9077					<0.001
Girl		55 (18.4)	1216 (53.6)	2267 (53.8)	1200 (52.2)	
Age	9077					<0.001
10 years old		9 (3.0)	72 (3.2)	176 (4.2)	39 (1.7)	
11 years old		68 (22.7)	313 (13.8)	774 (18.4)	142 (6.2)	
12 years old		66 (22.1)	410 (18.1)	792 (18.8)	258 (11.2)	
13 years old		57 (19.1)	374 (16.5)	650 (15.4)	491 (21.4)	
14 years old		42 (14.0)	356 (16.1)	542 (12.9)	420 (18.3)	
15 years old		19 (6.4)	307 (13.5)	538 (12.8)	436 (19.0)	
16 years old		24 (8.0)	259 (11.4)	439 (10.4)	323 (14.1)	
17 years old		14 (4.7)	169 (7.4)	299 (7.1)	189 (8.2)	
Family affluence	9077					<0.001
Lowest 20%		55 (18.4)	617 (27.2)	720 (17.1)	499 (21.7)	
Medium 60%		177 (59.2)	1229 (54.1)	2583 (61.4)	1357 (59.1)	
Highest 20%		67 (22.4)	424 (18.7)	907 (21.5)	442 (19.2)	
Residence	9077					<0.001
City		105 (35.1)	673 (29.6)	507 (12.0)	230 (10.0)	
Town		106 (35.5)	987 (43.5)	969 (23.0)	356 (15.5)	
Village		50 (16.7)	478 (21.1)	774 (18.4)	593 (25.8)	
Country		38 (12.7)	132 (5.8)	1960 (46.6)	1119 (48.7)	
Happiness with self	8979					<0.001
Happy with self		176 (59.5)	1150 (51.1)	2361 (56.7)	1126 (49.6)	
Body satisfaction	8933					<0.001
Satisfied		180 (61.2)	1216 (54.4)	1707 (58.9)	1236 (54.9)	
Self-rated health	9021					<0.001
Excellent health		123 (41.4)	630 (27.9)	1308 (31.3)	615 (26.9)	
Health symptoms	8145					<0.001
Multiple symptoms		80 (30.3)	777 (38.3)	1157 (30.6)	725 (35.1)	

Percentages are given for the respective columns. Association of the categorical variables with mode of transport were tested by Chi-square tests.

## Results

In total, 3.3% of the children (*n* = 299) reported cycling to and from school, while 25.0% (*n* = 2270) reported walking, 46.4% (*n* = 4210) commuted by private vehicle, and 25.3% (*n* = 2298) used public transport. The descriptive statistics are presented in Table 1 (sociodemographic and binary outcome variables) and Table 2 (continuous variables). All sociodemographic and outcome variables were associated with mode of transport. An age imbalance was observed across modes of transport. Younger children were more likely to cycle (peak at 11 years). For walking and use of private vehicles, the age peak was 12 years. Older children were more likely to use public transport (peak at 13 years).

**TABLE 2:** Descriptive statistics – continuous mental well-being outcome variables, Health Behaviour in School-aged Children study in Ireland, 2018.

	Total	Range	Cycling	Walking	Private vehicle	Public transport	*p*
			*M* (SD)	*M* (SD)	*M* (SD)	*M* (SD)	
Age	9077	10–17	13.42 (1.80)	13.99 (1.90)	13.76 (1.97)	14.49 (1.70)	<0.001
WHO-5	8798	0–100	60.29 (24.87)	55.82 (23.98)	59.89 (23.88)	55.84 (23.19)	<0.001
MHI-5	8750	0–100	25.68 (17.58)	31.94 (21.03)	27.80 (18.97)	30.97 (19.61)	<0.001
Life satisfaction	8869	0–10	7.56 (1.87)	7.21 (1.98)	7.62 (1.81)	7.24 (1.86)	<0.001

WHO-5: World Health Organization Five-item Well-being Index. MHI-5: Five-item Mental Health Inventory. Association of the continuous variables with mode of transport were tested by one-way variance of analysis.

### Continuous Variables

Estimated marginal means for WHO-5 Well-being Index, the MHI-5 scale, and the Cantril ladder across modes of transport and family affluence groups (controlled for area of residence) are presented in Table 3. Multivariate ANCOVAs are presented in Tables 4–6.

**TABLE 3:** Estimated marginal means for the World Health Organization Five-item Well-Being Index scores (*n* = 8798), the Five-item Mental Health Inventory scores (*n* = 8750) and life satisfaction (*n* = 8869) across modes of transport, Health Behaviour in School-aged Children study in Ireland, 2018.

Mode of transport	*n*	*M*	SD	95% CI
WHO-5^a^				
Cycling	290	58.44	1.80	54.92–61.96
Walking	2190	56.02	0.64	54.77–57.28
Private vehicle	4092	58.23	0.42	57.41–59.05
Public transport	2226	56.20	0.58	55.07–57.33
MHI-5^b^				
Cycling	284	29.01	1.12	26.81–31.21
Walking	2178	30.85	0.42	30.02–31.68
Private vehicle	4064	29.21	0.33	28.56–29.86
Public transport	2224	30.90	0.43	30.06–31.74
Life satisfaction^c^				
Cycling	295	7.46	0.13	7.20–7.72
Walking	2207	7.29	0.04	7.21–7.37
Private vehicle	4120	7.48	0.03	7.42–7.54
Public transport	2247	7.30	0.04	7.22–7.38

CI: Confidence interval. WHO-5: World Health Organization Five-item Well-being Index. MHI-5: Five-item Mental Health Inventory.

^a^ Controlled for gender, family affluence, area of residence, age, mode of transport × area of residence and mode of transport × age.

^b^ Controlled for gender, relative family affluence, area of residence and age.

^c^ Controlled for gender, relative family affluence, area of residence and mode of transport × gender.

**TABLE 4:** The impact of mode of transport on World Health Organization 5-item Well-being Index scores, controlled for gender, relative family affluence, area of residence and the interaction between mode of transport and area of residence (*n* = 8798), Health Behaviour in School-aged Children study in Ireland, 2018.

Predictor	Sum of squares	df	Mean square	*F*	*p*	Partial *η* ^2^	Power
Corrected model	610942.97	22	27770.13	55.49	<0.001	0.122	∼1.00
(Intercept)	1721911.62	1	1721911.62	3440.45	<0.001	0.282	∼1.00
Mode of transport	6838.65	3	2279.55	4.56	0.003	0.002	0.89
Gender	18865.43	1	18865.43	37.69	<0.001	0.004	∼1.00
Family affluence	15886.79	2	7943.39	15.87	<0.001	0.004	∼1.00
Area of residence	9392.68	3	3130.89	6.26	<0.001	0.002	0.97
Age	368078.42	1	368078.42	735.44	<0.001	0.077	∼1.00
Transport × Gender	9349.29	3	3116.43	6.28	<0.001	0.002	0.97
Transport × Area	9579.78	9	1064.42	2.13	0.024	0.002	0.89
Error	4391807.03	8775	500.49				

**TABLE 5:** The impact of mode of transport on the Five-item Mental Health Inventory scores, controlled for gender, relative family affluence, and area of residence (*n* = 8750), Health Behaviour in School-aged Children study in Ireland, 2018.

Predictor	Sum of squares	df	Mean square	*F*	*p*	Partial *η* ^2^	Power
Corrected model	381413.51	10	38141.35	110.40	<0.001	0.112	∼1.00
(Intercept)	131.76	1	131.76	0.38	0.537	<0.001	0.10
Mode of transport	5843.79	3	1947.93	5.64	0.001	0.002	0.95
Gender	120124.79	1	120124.79	347.70	<0.001	0.038	∼1.00
Family affluence	7053.22	2	3526.61	10.21	<0.001	0.002	0.99
Area of residence	31979.92	3	10659.97	30.86	<0.001	0.010	∼1.00
Age	162689.63	1	162689.63	470.91	<0.001	0.051	∼1.00
Error	3019171.68	8739	345.48				

**TABLE 6:** The impact of mode of transport on life satisfaction, controlled for relative family affluence, area of residence, and interaction of gender and mode of transport (*n* = 8869), Health Behaviour in School-aged Children study in Ireland, 2018.

Predictor	Sum of squares	df	Mean square	*F*	*p*	Partial *η* ^2^	Power
Corrected model	3238.61	13	249.12	78.56	<0.001	0.103	∼1.00
(Intercept)	19586.18	1	19586.18	6176.13	<0.001	0.411	∼1.00
Mode of transport	69.29	3	23.10	7.28	<0.001	0.002	0.98
Gender	16.09	1	16.09	5.07	0.024	0.001	0.62
Family affluence	161.07	2	80.53	25.40	<0.001	0.006	∼1.00
Area of residence	162.02	3	54.01	17.03	<0.001	0.006	∼1.00
Age	2189.62	1	2189.62	690.46	<0.001	0.072	∼1.00
Transport × Gender	59.55	3	19.85	6.26	<0.001	0.002	0.97
Error	28081.62	8855	3.17				

#### Perceived Well-Being

Mode of transport, on its own, had a significant but small effect on WHO-5 scores: *F* (3) = 21.71, *p* < 0.001, partial *η* ^2^ = 0.007. Gender, relative family affluence, area of residence, age, and two interaction parameters (mode of transport × area of residence and mode of transport × gender) significantly improved the model: *F* (22) = 55.49, *p* < 0.001, partial *η* ^2^ = 0.122 (Table 4). In the multivariate model, contribution of mode of transport was significant but marginal: *p* = 0.003, partial *η* ^2^ = 0.002.

Cyclists had the highest, and those who walked the lowest, estimated mean WHO-5 scores (Table 3). However, post-hoc tests revealed that only those who walked or used public transport had significantly lower well-being scores than those using private vehicles (*p* ≤ 0.020). There were no other significant differences in WHO-5 scores across transport modes.

#### Mental health Problems

Mode of transport, on its own, had a significant effect on MHI-5 scores: *F* (3) = 29.12, *p* < 0.001, but the effect size was small: partial *η* ^2^ = 0.010. Gender, relative family affluence, area of residence and age, but none of the interaction parameters, significantly improved the model: *F* (10) = 110.40, *p* < 0.001, partial *η* ^2^ = 0.112 (Table 5). In the multivariate model, contribution of mode of transport was significant but marginal: *p* = 0.001, partial *η* ^2^ = 0.002.

Cyclists had the lowest, and public transport users the highest, estimated mean of MHI-5 scores (Table 3). However, post-hoc tests indicated that only those who walked or used public transport had significantly poorer MHI-5 scores than those using private vehicles (*p* ≤ 0.013). There were no other significant differences between MHI-5 scores across transport modes.

#### Life Satisfaction

Mode of transport, on its own, had a significant effect on the Cantril ladder scores: *F* (3) = 32.94, *p* < 0.001, but the effect was small: partial *η* ^2^ = 0.011. Gender, relative family affluence, area of residence, age and the interaction between mode of transport and gender significantly improved the model: *F* (13) = 78,56, *p* < 0.001, partial *η* ^2^ = 0.103 (Table 6). The individual contribution of mode of transport, albeit significant, was marginal: *p* < 0.001, partial *η* ^2^ = 0.002.

In absolute value, those commuting by private vehicles had the highest, and those who walked the lowest, estimated mean of life satisfaction (Table 3), but the differences between the four groups were rather small. Post-hoc tests revealed that those who walked or used public transport had significantly lower life satisfaction than those who used a private vehicle (*p* = 0.001). There were no other significant differences in the life satisfaction across transport modes.

### Binary Variables

Table 7 presents the relative odds of the binary mental well-being outcomes across modes of transport, with cyclists as the reference group: CORs, and AORs – adjusted for gender, relative family affluence groups, area of residence and age – are presented alongside their 95% confidence intervals.

**TABLE 7:** Relative odds of being happy with self (*n* = 8879), satisfied with their body (*n* = 8933), reporting excellent health (*n* = 9021), and having multiple health symptoms (*n* = 8145) across modes of transport, Health Behaviour in School-aged Children study in Ireland, 2018.

Mode of transport	*n*	COR	*p*	95% CI	AOR	*p*	95% CI
**Happy with self**							
Cycling	296	1			1		
Walking	2251	**0.86**	0.004	0.78–0.95	1.01	0.871	0.92–1.11
Private vehicle	4162	0.95	0.346	0.87–1.05	1.02	0.644	0.93–1.12
Public transport	2270	**0.83**	0.001	0.75–0.93	0.99	0.787	0.89–1.09
**Body satisfaction**							
Cycling	294	1			1		
Walking	2236	**0.89**	0.018	0.81–0.98	0.97	0.595	0.88–1.08
Private vehicle	4152	0.96	0.419	0.88–1.06	1.00	0.932	0.90–1.10
Public transport	2251	**0.90**	0.030	0.81–0.99	0.98	0.681	0.88–1.08
**Excellent health**							
Cycling	297	1			1		
Walking	2257	**0.67**	<0.001	0.58–0.78	**0.80**	0.004	0.69–0.93
Private vehicle	4182	**0.76**	<0.001	0.66–0.87	**0.80**	0.002	0.69–0.92
Public transport	2285	**0.65**	<0.001	0.56–0.76	**0.74**	<0.001	0.64–0.87
**Multiple health complaints**							
Cycling	264	1			1		
Walking	2028	**1.26**	0.016	1.04–1.53	0.97	0.788	0.80–1.18
Private vehicle	3785	1.01	0.928	0.84–1.22	0.91	0.361	0.75–1.11
Public transport	2068	1.16	0.137	0.96–1.40	0.98	0.814	0.80–1.19

COR: crude odds ratio. AOR: odds ratio adjusted for family affluence, gender, area of residence and age. CI: confidence interval. For better readability, we highlighted significant odds ratios with bold letters.

#### Self-Esteem

Compared to cyclists, those who reported using public transport (COR = 0.83, 95% CI: 0.75–0.93) or walking (COR = 0.86, 95% CI: 0.78–0.95) had slightly but significantly lower odds for reporting that they are happy with the way they are. When controlled for sociodemographic variables, compared to cyclists none of these groups had significantly different odds to be happy with the way they are. Private vehicle users, either unadjusted or adjusted for sociodemographic variables, had similar odds for being happy with the way they are as cyclists.

#### Body Satisfaction

Compared to cyclists, those who walked (COR = 0.89, 95% CI: 0.81–0.98) or used public transport (COR = 0.90, 95% CI: 0.81–0.99) had slightly but significantly lower odds for being satisfied with their body. When controlled for sociodemographic variables, none of these groups had different odds to be satisfied with their body compared to cyclists. Private vehicle users, either unadjusted or adjusted for sociodemographic variables, had statistically similar odds for being satisfied with their body as cyclists.

#### Excellent Health

Compared to cyclists, all other transport groups had significantly lower odds for reporting excellent health: public transport users (COR = 0.65, 95% CI: 0.56–0.76), walkers (COR = 0.67, 95% CI: 0.58–0.78) and private vehicle users (COR = 0.76, 95% CI: 0.66–0.87). Controlling for sociodemographic variables did not change the pattern of these results: public transport users (AOR = 0.74, 95% CI: 0.64–0.87), walkers (AOR = 0.80, 95% CI: 0.69–0.93) and private vehicle users (AOR = 0.80, 95% CI: 0.69–0.92).

#### Multiple Health Complaints

Compared to cyclists, those who reported walking to and from school had slightly but significantly higher odds for having multiple complaints (COR = 1.26, 95% CI: 1.04–1.53). When controlled for sociodemographic variables, walkers’ odds for multiple health complaints were similar to that of cyclists. No significant patterns emerged, either unadjusted or adjusted for sociodemographic variables, for those who used a private vehicle or public transport to and from school.

## Discussion

Our results indicate that, in general, children who used cycling to commute to and from school report more positively on mental well-being indicators than those who use public transport on their way to and from school. Their scores, however, were not always statistically different from those using a private vehicle. Those who reported using a private vehicle or walking, usually scored in between the other two groups. When controlling for sociodemographic variables, the effect of mode of transport was either significant but marginal (for well-being, mental health problems and life satisfaction), or lost statistical significance (for self-esteem, body satisfaction, and health complaints). Cyclists, however, had a robust advantage compared to the other groups when they rated their health: they were significantly more likely to report excellent health, even after controlling for sociodemographic variables.

We observed a substantial imbalance across different modes of transport. Less than 4% of children reported cycling for the main part of their way to and from school, while more than 46% reported they traveled by a private vehicle. This imbalance might be attributed to transport infrastructure and its relationship with social inequalities in Ireland. The multivariate results indicate that both family affluence and area of residence (urban or rural) have some impact on the association between mode of transport and mental well-being. Children who are allowed and encouraged by their parents to use bicycles to commute to school are likely to be living relatively close to their school, and it seems reasonable to assume that the infrastructure is also deemed safe by the parents (e.g., there is a separate bike lane, or car traffic on the roads is low). Similar factors, such as social cohesion within the local community may also influence parents’ decisions on allowing their children to actively travel to and from school (13). The main factor which seems to be associated with mental well-being, was age – to such an extent that for most outcomes it obscured the effect of mode of transport. When we had conducted the analyses without controlling for age, the models controlled for other sociodemographic variables were very similar to the baseline models. Age distribution across modes of transport was imbalanced: younger children were more likely to report cycling to and from school, while children walking or using a private vehicle on the way to and from school were somewhat older; children who used public transport were the oldest. From the data we cannot infer the reason for this imbalance. A potential explanation is that in Ireland, many children attend primary schools relatively close to their homes, but post-primary schools are larger and generally further away – multiple primary schools act as ‘feeder’ schools to post-primary schools. Therefore younger children, attending primary schools, may have more opportunity to cycle to and from school, while students at post-primary schools may have to use public transport. There is evidence from international (27) as well as Irish (28) studies that mental well-being in children and adolescents decreases with age, medium and late adolescents becoming more vulnerable to low life satisfaction and depressive symptoms than early adolescents.

Children who use public transport to and from school reported poorer mental well-being outcomes than the other groups. Beside age, this may also be influenced by other family or contextual factors, such as low socio-economic status (although the models adjusted for family affluence but not for age rendered patterns similar to the baseline models). Young people who live in socio-economically disadvantaged neighbourhoods have lower rates of physical activity (16).

We found that relatively few children cycle to and from school, and only around one fifth (18.2%) of them are girls. Boys and girls were roughly equally likely to report using the other three modes of transport. We cannot infer the reasons for this gender imbalance in cycling from our data. However, a qualitative study of girls from New Zealand demonstrated that some adolescent girls report feeling self-conscious in cycle clothing, and that the perceived lack of femininity of cycling discouraged them (5). Girls may have greater concerns about dangers related to cycling and have lower self-perceived cycling ability and skills (e.g., knowledge of local cycle routes and bicycle maintenance). Girls’ parents may also be more worried and restrictive about their daughters’ cycling. Image, in relation to their maturity and femininity, desire not be seen doing physical exercise, and the gendering of cycling have been linked to girls’ decisions to not use bicycles (5).

There are other important factors that may impact the relationships between mode of transport to school and mental-wellbeing. One such factor is experiencing peer violence. Among schoolchildren participating in the 2009/2010 HBSC study in Canada, it was demonstrated that bullying victimization was significantly more frequent in children who used active transportation compared to their peers who used other ways of transportation (OR = 1.26) (29). Given the ample evidence for the association of bullying victimization and negative mental well-being outcomes in adolescence (30), we anticipate that bullying may mediate or moderate the link between modes of transportation between school and home and mental well-being in young people.

Almost half of the children in our study reported commuting via private vehicles, illustrating that Ireland has a car-centred traffic culture. Children in Ireland (31) travel between home and school at peak traffic flow periods, which also increases local traffic congestion and pollution. Children in rural areas were more likely to report using a private vehicle, which may be attributed to the fact that public transport largely serves urbanized areas, and rural public transport is scarce. Lack of accessible public transport in rural Ireland is understood as a form of social exclusion (32). For many rural children whose parents don’t have a car or cannot give them a lift, a school bus is the only option to commute between home and school (31). This may also explain our findings that public transport users reported the poorest mental well-being outcomes. Another environmental factor determining which mode of transport is used by children is the distance between home and school (29). Further investigation is needed to better understand which factors influence children’s and their parents’ preferred modes of transport in Ireland, why using public transport is associated with poorer mental well-being, and why cycling shows a gendered pattern that is unfavourable for girls.

Relative family affluence and area of residence had a relatively small but significant influence on the associations of modes of transport and mental well-being outcomes. There are other environmental and contextual factors which are documented to influence active travel to school. In a Canadian study with 397 schools (33), school-level factors (policies and infrastructural investments such as theft-proof bicycle racks) as well as attributes of the environment (rubbish in the streets, crime, and substance abuse) had an impact on children’s active travel. A large portion (42%) of schools were located on high-speed roads not suitable for active travel and 14% lacked a sidewalk leading to the school. These findings suggest that in addition to family affluence and area of residence, attributes of neighbourhoods (e.g., how safe the area or route to school is for cycling, social cohesion, norms around different modes of transport) should be considered in future investigations. Irish children themselves have recognized multiple determinants of active travel, and their complexity, and highlighted the need for a multi-sectoral approach to this issue (15).

This study is strengthened by use of a nationally representative and adequately powered sample. Moreover, internationally comparable measures of mental well-being were used. However, there are some limitations. The measures of mental well-being used in our study are general rather than specific. Other confounder variables (such as bullying victimization, family violence, family or peer support, and distance between school and home) may have an impact on the association between mental well-being and modes of transport. One may argue that we should have included those children (around 12% of the sample) who use discordant modes of transport to and from school. While excluding their responses meant a data loss, if their mental well-being had been associated with mode of transport, including children using discordant modes of transport would have confounded the results. Further studies, using structural equation modeling, are needed to situate mode of transport between school and home as a determinant of adolescent mental well-being.

Nonetheless, our results support the argument that cycling is associated with better self-perceived health, which gives further justification to policy actions to promote cycling in children, such as those reported by the Irish Green-Schools Transport initiative (8). Encouraging girls and older adolescents to use bicycles to commute between school and home is likely to have a positive indirect effect on their physical and mental well-being.

Active transport is increasingly recognized as a way to advance multiple agendas, including improving individual physical activity, traffic management, and environmental protection (9). It seems worthwhile to invest in developing cyclist-friendly infrastructures, training on roads and cycling safety for children and families. The Irish National Cycle Policy Framework (14) highlighted the need for “a mandatory national cycling proficiency program for all school children in Irish schools starting at primary level and continuing in a graduated manner through to secondary level” (p. 35). To our knowledge, this program has not been implemented since the publication of the framework, though the recent program for Government has pledged an increase of expenditure on cycling from 2 to 10% of the national transport budget. Our results indicate that such policy actions could be successful, but attention to current and potential social, environmental and infrastructural inequalities must be incorporated.

## Data Availability

The datasets analysed in this study can be accessed at the webpage of the HBSC Data Management Centre (https://www.uib.no/en/hbscdata), in accordance with the HBSC data access policy. Requests to access these datasets should be directed to the HBSC International Coordinating Centre, iccadmin@hbsc.org.
